# Extensive transgressive gene expression in testis but not ovary in the homoploid hybrid Italian sparrow

**DOI:** 10.1111/mec.16572

**Published:** 2022-07-04

**Authors:** Homa Papoli Yazdi, Mark Ravinet, Melissah Rowe, Glenn‐Peter Sætre, Caroline Ø. Guldvog, Fabrice Eroukhmanoff, Alfonso Marzal, Sergio Magallanes, Anna Runemark

**Affiliations:** ^1^ Department of Biology Lund University Lund Sweden; ^2^ School of Life Sciences, University of Nottingham Nottingham UK; ^3^ Department of Animal Ecology Netherlands Institute of Ecology (NIOO‐KNAW) Wageningen The Netherlands; ^4^ Department of Biosciences, Centre for Ecological and Evolutionary Synthesis University of Oslo Oslo Norway; ^5^ Department of Anatomy, Cellular Biology and Zoology University of Extremadura Badajoz Spain; ^6^ Department of Wetland Ecology Doñana Biological Station (EBD‐CSIC) Seville Spain

**Keywords:** gene expression, hybridization, mito‐nuclear genes, testis and transgressive expression

## Abstract

Hybridization can result in novel allelic combinations which can impact the hybrid phenotype through changes in gene expression. While misexpression in F_1_ hybrids is well documented, how gene expression evolves in stabilized hybrid taxa remains an open question. As gene expression evolves in a stabilizing manner, break‐up of co‐evolved *cis*‐ and *trans*‐regulatory elements could lead to transgressive patterns of gene expression in hybrids. Here, we address to what extent gonad gene expression has evolved in an established and stable homoploid hybrid, the Italian sparrow (*Passer italiae*). Through comparison of gene expression in gonads from individuals of the two parental species (i.e., house and Spanish sparrow) to that of Italian sparrows, we find evidence for strongly transgressive expression in male Italian sparrows—2530 genes (22% of testis genes tested for inheritance) exhibit expression patterns outside the range of both parent species. In contrast, Italian sparrow ovary expression was similar to that of one of the parent species, the house sparrow (*Passer domesticus*). Moreover, the Italian sparrow testis transcriptome is 26 times as diverged from those of the parent species as the parental transcriptomes are from each other, despite being genetically intermediate. This highlights the potential for regulation of gene expression to produce novel variation following hybridization. Genes involved in mitochondrial respiratory chain complexes and protein synthesis are enriched in the subset that is over‐dominantly expressed in Italian sparrow testis, suggesting that selection on key functions has moulded the hybrid Italian sparrow transcriptome.

## 1 INTRODUCTION

Hybridization between recently diverged species is an important evolutionary phenomenon resulting in novel gene combinations that become available to selection (Abbott et al., 2013; Mallet, 2005; Runemark et al., 2019; Taylor & Larson, 2019). While bringing together diverged parental alleles in hybrids can lead to reduced hybrid fitness (Dobzhansky, 1936; Muller, 1942), it is now clear that hybridization is frequent and contributes to novel adaptive phenotypes (Marques et al., 2019; Runemark et al., 2019; Taylor & Larson, 2019). Despite the growing body of evidence documenting admixed genomes, it is not clear how such intermediate genomes give rise to transgressive hybrid phenotypes (Rieseberg et al., 1999). Gene expression is the key intermediate step between genotype and phenotype, and divergence in the regulation of gene expression is thought to play an important role in phenotypic evolution (Hodgins‐Davis et al., 2015; Rest et al., 2013). Despite this, we lack an understanding of how gene expression evolves and contributes to novel phenotypes in hybrid taxa.

Gene expression evolves in a stabilizing manner, where regulatory elements accumulate mutations that keep gene expression at an optimum level for physiological functions (Coolon et al., 2014; Gilad et al., 2006; Hodgins‐Davis et al., 2015). Specifically, changes in distal, *trans*‐regulatory elements are compensated for by changes in proximate, *cis*‐regulatory elements (Landry et al., 2005). Such compensatory evolution means that as hybridization breaks up co‐inheritance of regulatory elements, hybrids may experience novel combinations (Landry et al., 2005; Renaut et al., 2009). The novel combinations of regulatory elements arising during hybridization has the potential to lead to transgressive gene expression that transcends the range of parental expression profiles.

Hybrids resulting from strongly divergent taxa may exhibit extensive levels of transgressive expression arising from uncoupling of co‐evolved *cis‐* and *trans‐*regulatory elements in F_1_ hybrids (Coolon et al., 2014; Haerty & Singh, 2006; McManus et al., 2010). In post‐F_1_ generation hybrids, transcriptomes show a higher level of transgressive expression due to uncoupling of regulatory elements in the process of recombination. In the lake white fish *Coregonus clupeaformis* for example, F_2_ hybrids showed a higher level of nonadditive inheritance in gene expression compared to F_1_ hybrids (Renaut et al., 2009). An additional line of evidence of a nonlinear relationship between genetic composition and gene expression comes from recent studies on allopolyploid plant taxa, hybrid taxa with doubled chromosome number. Both preferential expression of one of the parental genomes (Edger et al., 2017) and tissue‐dependent parental dominance in expression pattern (Kryvokhyzha et al., 2019) have been documented in allopolyploids. Jointly, the transgressive expression in experimental hybrids and nonlinear relationship between genomic composition and gene expression similarity suggest that gene expression will not be proportional to the parental contributions to the genome. Whether transgressive patterns of gene expression are found in homoploid hybrid taxa, hybrids without an increase in ploidy, is unknown.

**FIGURE 1:**
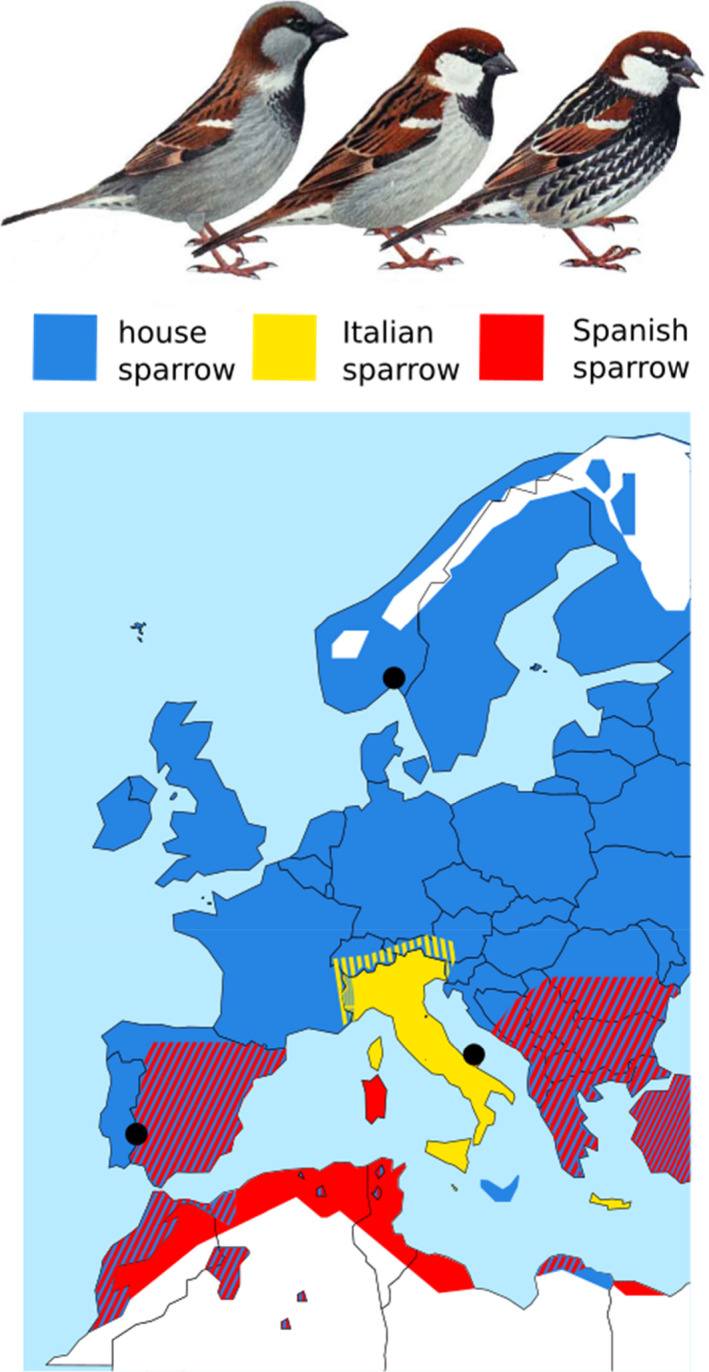
Species distribution and sampling locations. Top: Illustrations of male plumage patterns in house, Italian and Spanish sparrows modified from Svensson et al. (1999). Bottom: Distribution map of house, Italian and Spanish sparrows throughout Europe and northern Africa (Summers‐Smith, 1988). This picture is modified from Elgvin et al. (2017)

Here, we utilize a unique study system to investigate the nature of gene expression in a stabilized hybrid taxon, the homoploid hybrid Italian sparrow (*Passer italiae*), where thousands of generations of selection have resulted in a stable genome composition. The Italian sparrow arose from hybridization between the house sparrow (*Passer domesticus*) and Spanish sparrow (*Passer hispaniolensis*) (Figure 1) (Elgvin et al., 2017; Hermansen et al., 2011; Hermansen et al., 2014; Trier et al., 2014). The parent species diverged ~0.85 million years ago (Ravinet et al., 2018) and the Italian sparrow originated within *~*5800 years, probably as the house sparrow expanded its range into Europe and hybridized with the Spanish sparrow (Ravinet et al., 2018). Genome‐wide, the Italian sparrow is intermediate between the parental species, the house and Spanish sparrow. The proportion of house sparrow ancestry varies across different Italian sparrow populations (Runemark, Trier, et al., 2018), with mainland Italian sparrow sampled close to the region Guglionesi having about 62% house sparrow and 38% Spanish sparrow ancestry (Elgvin et al., 2017). The global average level of genetic differentiation is higher between parents (house–Spanish *F*_ST_ = 0.33) than between Italian sparrow and each parental species (house–Italian *F*_ST_ = 0.18, Spanish–Italian *F*_ST_ = 0.25). Almost every Italian sparrow sampled has house sparrow mitochondrial DNA (mtDNA) (Elgvin et al., 2017; Runemark, Trier, et al., 2018). Populations of the Italian sparrow have some regions of the genome that are inherited from one parent species, especially the Z chromosome, with an over‐representation of for example house sparrow mito‐nuclear alleles (Runemark, Trier, et al., 2018). However, large parts of the genome also have segregating alleles from both parent species (Runemark, Trier, et al., 2018). This system provides a unique possibility to test to what extent a homoploid hybrid species has diverged in gene expression from the parent species after thousands of generations. In this study, we compared the gene expression profiles of the gonads of wild individuals of Italian sparrow to gene expression of the parental species to address if gene expression in Italian sparrows is intermediate to that of its parent species. At the same time, we opportunistically compared gene expression profiles to experimental F_1_ hybrid crosses (house × Spanish) bred in captivity. Thereby, we test whether gene expression reflects the intermediate genome composition of the hybrid Italian sparrow, or if it is transgressive for specific gene categories.

## 2 MATERIALS AND METHODS

### 2.1 Sampling, RNA extraction, library preparation and sequencing

We compared the gene expression of wild Italian sparrows to wild individuals of its two parent species, the house and Spanish sparrow. Although the species differ in their distribution and phenology, all birds were sampled during the breeding season when birds were actively breeding (Appendix S1) at their respective locations. Spanish sparrows were sampled near Olivenza, Spain (38°40′56″N, 7°11′17″W), in March 2016, house sparrows in Oslo, Norway (59°55′2″N, 10°46′11″E), in May 2016 and Italian sparrows from Masseria Montanari in the Gargano peninsula (41°54′36.8″N, 15°51′13.0″E) in May 2016 (Figure 1). All birds were trapped using mist nets and placed in a soft cotton bird bag until processing. All birds were killed by cervical dislocation, and gonads (ovary and left testis) were harvested immediately after death. Sampling was performed between 10 a.m. and 4 p.m., with individuals from each of the species being sampled over most of the day, in order to avoid biases in sampling time. The gonads were stored in RNAlater, and immediately stored at −80°C, with extractions later performed at the University of Oslo using the Qiagen miRNeasy micro kit. Samples were prepped with TruSeq Stranded mRNA library prep from Illumina on an automated Perkin Elmer Sciclone NGSx liquid handler and sequenced in the Norwegian Sequencing Center in Oslo, as paired‐end (2 × 150 bp) on the Illumina Hiseq4000. The number of reads obtained per each sample is reported in Table S1.

### 2.2 Read mapping, quality check and counting

We mapped reads from samples of all three groups (testis sample size: house sparrow = 5, Spanish sparrow = 5, Italian sparrow = 5; ovary sample size: house sparrow = 5, Spanish sparrow = 3 and Italian sparrow = 5) to the house sparrow reference genome using star2 version 2.7.2b (Dobin et al., 2013) with default parameters and the house sparrow gene annotation as reported in Elgvin et al. (2017). Over 90% of the reads mapped successfully (Table S1). The house sparrow general feature format (GFF) file contained 14,734 annotated genes, of which 92.52% were anchored to chromosomes and 7.48% were located on unanchored scaffolds. Of the genes anchored to chromosomes, 12,595 were annotated on autosomes and 598 were on the Z chromosome (Table S2). To ensure homogeneity in quality across our samples, we used rseqc (Wang et al., 2012) to measure RNA integrity at the transcriptome level for each sample by calculating the Transcript Integrity Number (TIN). The median TIN scores across samples ranged between 73.7 and 85.9 (Table S3). We counted the number of reads mapping to the house sparrow gene features using htseq version 0.9.1 (Anders et al., 2015). Read counting was performed for all reads with minimum quality score of 30 and configured to handle reverse‐stranded sequencing data, with the parameter controlling for overlapping gene features set to union. We created a dendrogram to show sample relationships. The main split was clearly between tissue types, namely testis and ovary (Figure S1).

### 2.3 Differential gene expression analysis

We analysed the raw read counts obtained from htseq‐count using the R package deseq2 (Anders & Huber, 2010; Love et al., 2014). We first tested genes for differential expression between house and Spanish samples. We then compared each of the parents to the Italian sparrow. To check whether the deseq2 model is a good fit to our data, we plotted the dispersion estimates as a function of mean of normalized counts (Figures S2 and S3). The dispersion curve for testis samples showed a good fit with a general scatter of data around the fitted curve and decreasing dispersion values with increasing mean expression levels (Figure S2). For ovary, the overall trend of decreasing dispersion with increasing mean value was observed for all comparisons. In particular, the comparison between house and Italian sparrow showed a good fit, although there was a larger scatter of dispersion values with higher means in the comparisons involving Spanish ovary (house–Spanish and Spanish–Italian), indicating a higher within‐group dispersion in the Spanish samples (Figure S3). We prefiltered data to a minimum of total read counts of 10 across at least half of the samples in each pairwise comparison. To generate more accurate estimates of log_2_ fold change (LFC) for genes with low count number or large dispersion, we shrank LFC estimates. We considered genes to be differentially expressed if they showed a false discovery rate (FDR) *p*_adj_ < .05 and shrunken LFC > 0.32. We used a hypergeometric test in R with phyper() function to examine the over‐representation of Z‐linked genes among differentially expressed genes using the number of differentially expressed genes on Z and autosomes and the number of total genes with measured expression on each chromosome category. All statistical analyses were completed using R version 4.0.2 (R Core Team, 2020).

### 2.4 Classification of gene expression inheritance

The mode of inheritance for differentially expressed genes was determined following McManus et al. (2010). We normalized gene expression using the median of ratios method in deseq2 (Love et al., 2014). Genes whose total expression in Italian sparrow deviated significantly more than 1.25‐fold (LFC > 0.32) from that of either parent were considered to have nonconserved inheritance. These genes were classified as having additive, dominant, under‐dominant or over‐dominant inheritance, based on the magnitude of the difference between total expression in the Italian sparrow and in each parental species. Genes for which expression in the Italian sparrow was less than for house sparrows and greater than for Spanish sparrow (or vice versa) were classified as additive; genes for which expression in Italian sparrow was similar to one of the parents were classified as dominant; and genes for which expression in Italian sparrow was either greater than or less than both parent species were classified as over‐dominant and under‐dominant (transgressive), respectively (Figure 3a,b).

### 2.5 Functional annotation and enrichment analysis

Gene Ontology (GO) analysis was done for differentially expressed genes in each pairwise comparison. GO functional annotations and gene descriptions were obtained for protein sequences from the entire house sparrow protein set as described in Rowe et al. (2020) using pannzer (Koskinen et al., 2015). Functional enrichment of GO terms present in the set of differentially expressed genes relative to the background consisting of all genes expressed and tested for differential expression was performed using clusterprofiler (Yu et al., 2012) and significant enrichment was determined at an FDR, 0.05. Interactions among proteins with significant GO terms were predicted using string version 11 (Szklarczyk et al., 2019) using “Experiment,” “Databases” and “Co‐expression” as interaction sources. We set the minimum required interaction score to the highest confidence (0.9) and used only the query proteins to build the interaction network after removing the disconnected nodes in the network. The obtained network was then exported to cytoscape (Shannon et al., 2003) and major representations of biological processes were detected using cluego (Bindea et al., 2009) with *p*_adj_ < .01.

### 2.6 Analysis of experimental hybrids

Although our study was designed to allow the comparison of wild Italian sparrows to wild individuals of its two parent species, the house and Spanish sparrow, we also capitalized on the availability of captive‐bred experimental F_1_ hybrids produced by crossing house sparrow females and Spanish sparrow males (eight ovary, five testis) (details about the breeding of experimental F_1_ hybrids are presented in Appendix S1, Figure S4). As experimental hybrids were bred in an area where house and Spanish sparrows are sympatric for at least part of the year (Olivenza, Spain; see Figure 1) and correct identification of females can be challenging, we performed a post‐sampling genomic analysis of all samples (Appendix S1). The mtDNA of experimental F_1_ hybrids were correctly grouped with house sparrow (Figure S5). However, the F_1_ hybrids did not show the expected equal ancestry ratio from each parental species and a principal components anslysis (PCA) based on the Z chromosome of F_1_ females showed clustering with the Z chromosome from house sparrow contrary to the expectation (Figures S6 and S7). We additionally used independent data sets from whole genome resequencing studies of different *Passer* species to perform PCA to verify the placement of each of our samples (Figure S8). We therefore acknowledge that the comparison between these experimental hybrids and the other species sampled in our study is suboptimal. Nonetheless, because the comparison of the stabilized homoploid hybrid Italian sparrow and artificial early generation hybrids between house and Spanish sparrows provides an interesting contrast, we decided to retain the experimental hybrid samples while clarifying the limitations to how these findings can be interpreted. All gene expression analyses were carried out in these experimental hybrids in the same way as described for the Italian sparrow samples.

## 3 RESULTS

### 3.1 Differential expression between the parental species

Gene expression divergence in gonads between the parental species was strongly asymmetric with testis showing a more conserved pattern of expression compared to ovary (Table 1). In total, 135 genes (1.16%) testis and 1382 genes (11.65%) in ovary were significantly differentially expressed. Of the differentially expressed genes, 60.7% of genes in testis and 69.3% of genes in ovary were upregulated in the Spanish sparrow. A significantly higher proportion of genes that were differentially expressed in testis were located on the Z chromosome (24 genes, hypergeometric test, *p* < 1.82–08). We found no evidence for functional enrichment among differentially expressed genes in parental species for either testis or ovary.

**TABLE 1:** Number of differentially expressed genes and log_2_ fold change (LFC) with *p*_adj_ < .05^**a**^ and LFC^**b**^ > 0.32

	Testis			Ovary		
Comparison	Significant	LFC > 0	LFC < 0	Significant	LFC > 0	LFC < 0
Spanish–house	135	82	53	1382	958	424
Italian–house	3536	1962	1574	22	13	9
Italian–Spanish	3581	1779	1802	1508	394	1114

^a^ Results with padj < .01 are reported in Table S4.

^b^ LFC, Log Fold Change.

### 3.2 Differential expression in Italian sparrows compared to the parental species

**FIGURE 2:**
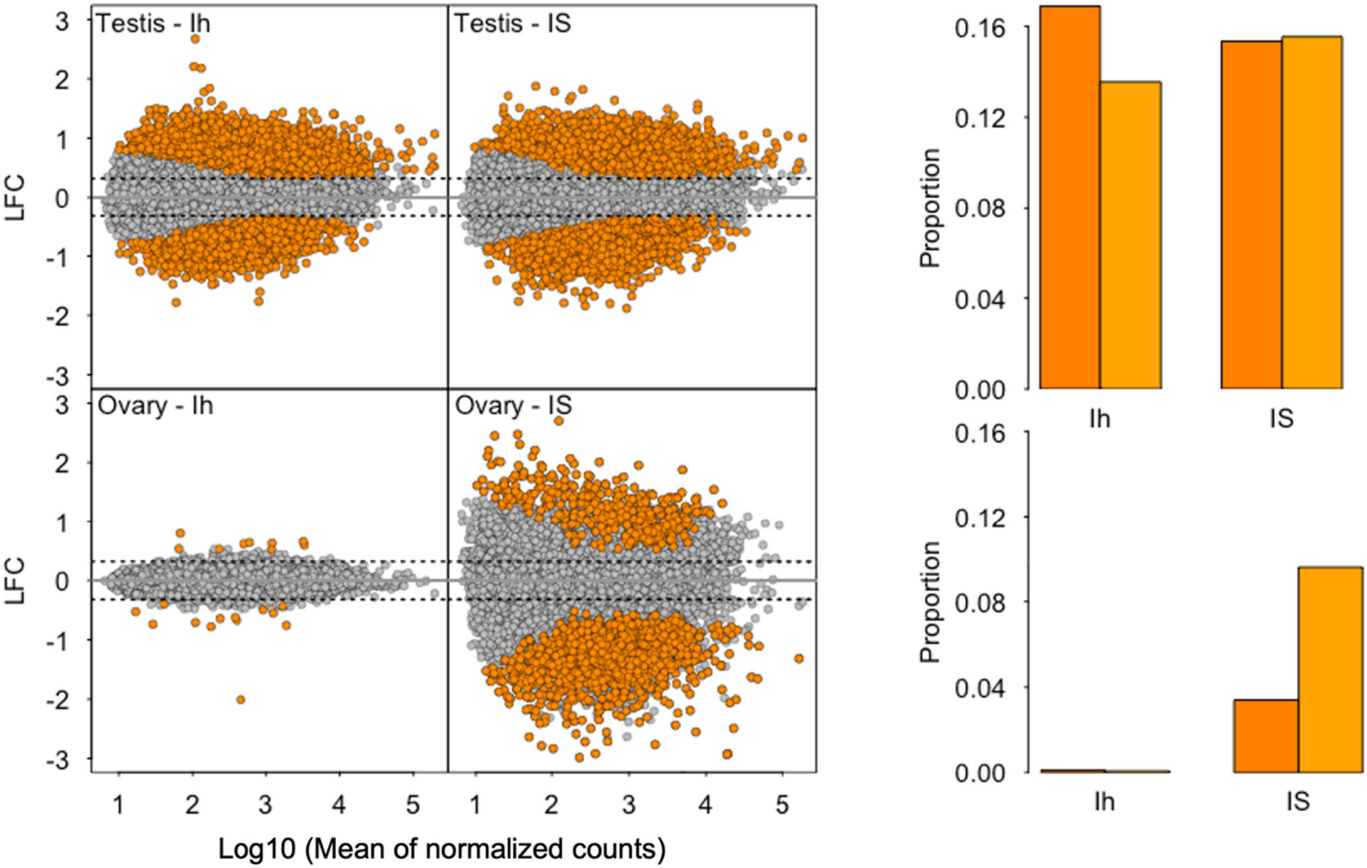
Gene expression in Italian sparrow in comparison to house (Ih) and Spanish (IS) for testis (top) and ovary (bottom). Log_2_ fold change (LFC) is plotted as a function of the mean of normalized counts. Significantly differentially expressed genes are shown in orange and conserved genes in grey. Bar plots summarize the proportion of up‐ and down‐regulated genes. Dark orange: Up‐regulated, light orange: Down‐regulated

In contrast to the intermediacy of the Italian sparrow genome, Italian sparrow testis gene expression differed more in comparison to the parental species than the parental species differed from each other (Table 1). Specifically, approximately one‐third of the testis transcriptome was differentially expressed in Italian sparrow compared to both parental species, whereas ovary expression was similar to that of the house sparrow (Table 1, Figure 2). In the Italian sparrow testis, 3536 genes (30.45%) and 3581 genes (30.9%) were differentially expressed in comparison to house and Spanish sparrow, respectively. For Italian sparrow ovary, only 22 genes (0.18%) differed from the house sparrow whereas 1508 genes (12.63%) were differentially expressed compared to the Spanish sparrow. Significant over‐representation of Z‐linked genes among the differentially expressed genes was detected in testis, but only in the comparison to Spanish sparrow (196 of the 3581 genes, hypergeometric test, *p* = .006). Most genes in both testis and ovary were up‐regulated compared to house sparrow and down‐regulated compared to Spanish sparrow (Chi‐squared test *p* = 1.05e‐06 in testis and 0.001 in ovary) (Figure 2).

Genes that were differentially expressed in Italian sparrow testis were enriched for similar biological processes in comparison to both parent species. Primarily, functions involving protein synthesis and mitochondrial gene expression and function were overrepresented. Comparisons between the Italian sparrow and the house sparrow yielded 27 significant GO categories (Table S5), with top biological processes involved in viral transcription, SRP‐dependent cotranslational protein targeting to membrane, translation, ribosome and mitochondrial respiratory chain complex I assembly. In comparisons between the Italian sparrow and the Spanish sparrow, the 15 enriched GO terms reflected similar biological processes, with ribosome as the top GO term (Table S6). In ovary, six GO terms were detected in the comparison to house sparrow, with sodium ion transmembrane transporter activity as the top term (Table S7) and one GO term, negative regulation of protein phosphorylation, detected in the comparison to Spanish sparrow (Table S8).

### 3.3 Classification of inheritance patterns of gene expression

**FIGURE 3:**
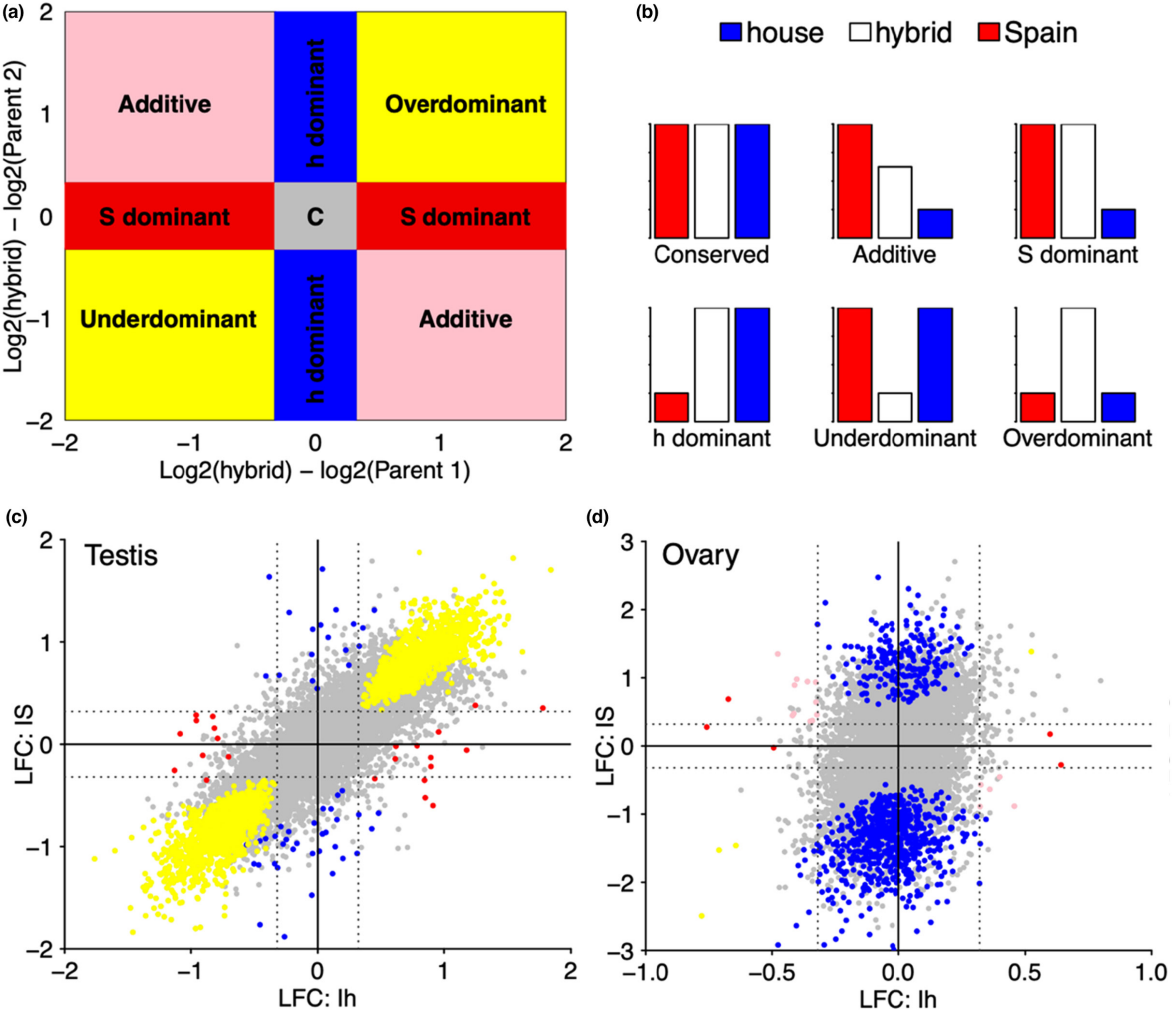
Inheritance pattern of gene expression in testis and ovary in Italian sparrows. (a,b) Schematic figures representing the classification of inheritance patterns: House sparrow (h), Spanish sparrow (S). (c) Scatter plot showing shrunken log_2_ fold change (LFC) in testis between the Italian sparrow with the house sparrow (Ih) on the *x*‐axis and with Spanish sparrow on the *y*‐axis (IS), respectively. (d) Scatter plot showing shrunken log_2_ fold change (LFC) in ovary between the Italian sparrow with the house sparrow on the *x*‐axis and with Spanish sparrow on the *y*‐axis, respectively. Grey points in each graph depict the total number of genes studied for gene expression with those coloured representing the ones significantly different from parental species to be divided into each of the inheritance categories (conserved: Grey, additive: Pink, house‐dominant: Blue, Spanish‐dominant: Red, transgressive [over‐dominant and under‐dominant]: Yellow). Grey dotted lines indicate the log_2_ fold‐change threshold of 0.32 used for classification of inheritance categories

**FIGURE 4:**
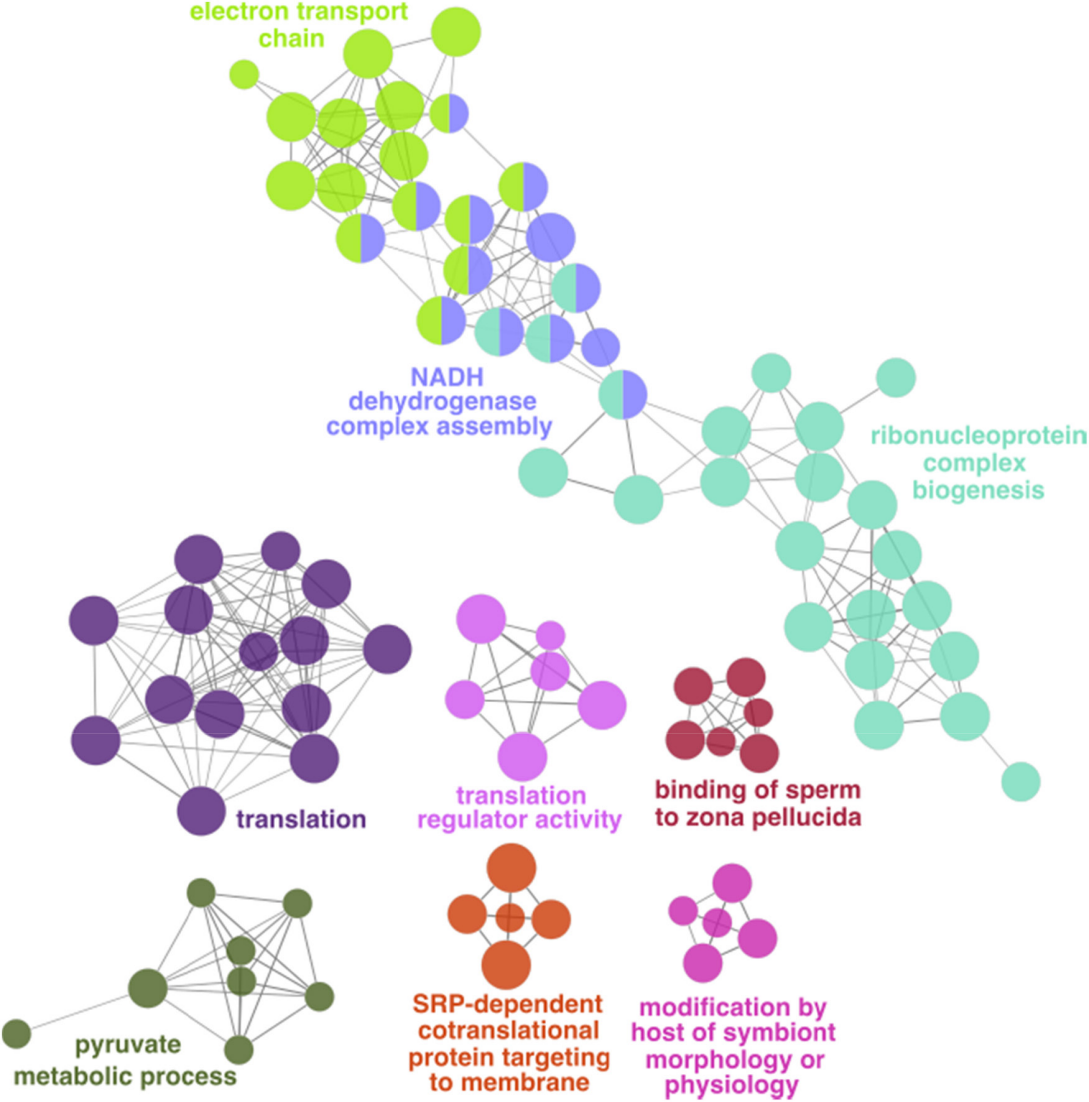
Gene ontology network represented in over‐dominant genes in testis of Italian sparrow. Different colours depict significantly different biological processes. Circle size represents adjusted *p* value for each node with all *p*_adj_ < .01 where node size represents the term enrichment significance

Gene expression was generally conserved in Italian sparrow (Table 2), but in the testis of Italian sparrow, 2611 genes (22.71% of genes evaluated for inheritance pattern) showed a nonconserved pattern of inheritance with transgressive expression and house sparrow‐dominant being the two largest categories (Figure 3c). Transgressively expressed genes in Italian sparrow comprised 22% of the testis transcriptome tested for inheritance. Over 96% of genes with a nonconserved pattern of inheritance in Italian sparrow ovary had a house sparrow‐dominant pattern of expression (Figure 3d). In contrast to the high incidence of transgressive expression observed in Italian sparrow testis, only four genes (0.028%) were transgressively expressed in ovaries. Over‐dominant genes in Italian sparrow testis were enriched for functional categories involved in protein synthesis, mitochondrial protein complex and gene expression and binding of sperm to zona pellucida (Table S10). We found a gene network with significant protein–protein interaction (PPI Enrichment: 1.0E‐16) among the over‐dominant genes in Italian sparrow (Figure 4). A different set of genes was under‐dominant in Italian sparrow, with functions including regulation of cellular component size and negative regulation of neuron projection development.

**TABLE 2:** Number and percentage of genes in each inheritance category for Italian sparrow^**a**^

	Testis	Ovary
Inheritance category		
Conserved	8883 (77.30%)	10,889 (92.26%)
Additive	2 (0.02%)	19 (0.16%)
house dominant	54 (0.47%)	895 (7.58%)
Spanish dominant	25 (0.22%)	7 (0.06%)
Under‐dominant	1205 (10.47%)	3 (0.02%)
Over‐dominant	1325 (11.53%)	1 (0.008%)

^a^ Inheritance classification for differentially expressed genes with *p*_adj_ < .01 is reported in Table S9.

### 3.4 Patterns of expression in experimental hybrids

The overall magnitude of differences in gene expression in the experimental hybrid compared to both parent species was much lower than for the Italian sparrow (Figure S9). In testis, 106 genes (0.9%) and 263 genes (2.25%) were differentially expressed compared to house and Spanish sparrows, respectively. In ovary, 30 genes (0.25%) and 140 genes (1.15%) showed a significant difference in expression compared to the house and Spanish sparrows, respectively. Moreover, in the experimental hybrid testis, only 0.37% of the genes were transgressively expressed (Table S11). Gene expression in experimental hybrid ovary also was more conserved in relation to the parent species compared to that of the Italian sparrow, with the two largest categories of inheritance being additive and house sparrow‐dominant (Table S11). Additional results from the experimental hybrids are presented in Appendix S1 and Tables S12–S15.

## 4 DISCUSSION

This study presents, to the best of our knowledge, the first evidence for transgressive gene expression in a wild homoploid hybrid species. The strongly divergent gene expression profile in Italian sparrow testis highlights the potential of intermediate hybrid genomes to rapidly evolve through altered regulation of gene expression. About 22% of testis genes tested for inheritance (2530 genes) in Italian sparrow showed a transgressive expression pattern, while this level was <0.03% in the ovaries (four genes). Transgressive gene expression, despite conserved parental expression, could arise in later generation hybrids due to the uncoupling of co‐evolved *cis*‐ and *trans*‐regulatory elements through recombination (Landry et al., 2005; Signor & Nuzhdin, 2018; Takahasi et al., 2011). There are, however, few studies of gene expression in post‐F_1_‐hybrids and transgressive expression is commonly referred to “mis‐expression” in early generation hybrids (Signor & Nuzhdin, 2018). Here, we show that the homoploid hybrid Italian sparrow has achieved a 26‐fold stronger divergence in testis gene expression during ~5800 years of evolution following hybridization than the parents have accumulated during ~0.85 million years (Ravinet et al., 2018). This might imply that sorting of parental regulatory elements following hybridization can produce novel expression phenotypes from intermediate genomes.

Hybrid incompatibilities leading to reproductive isolation due to novel, untested genetic combinations are most commonly thought to occur when parental species are fixed for different alleles (Cutter, 2012). However, transgressive expression patterns in the hybrids of more recently diverged species pairs can also arise either due to polymorphic incompatible loci (Cutter, 2012) or physiological responses to hybrid dysfunction (Barreto et al., 2015). In interpopulation hybrids at F_2_ or later generation of a copepod species (*Tigriopus californicus*), transgressive gene expression mainly reflected physiological responses in hybrids (Barreto et al., 2015). Therefore, given the relatively low divergence between house and Spanish sparrow, transgressive expression in the Italian sparrow could also be due to bringing together polymorphic *cis*‐ and *trans*‐regulatory variants from the parental species. Alternatively, transgressive gene expression may arise from lineage‐specific mutations that have been favoured by selection in Italian sparrow. Each of these hypotheses can be evaluated in future work by studying the optimal F_1_ and F_2_ hybrids and their parental individuals to identify *cis‐* and *trans*‐regulatory variants.

Gene expression was conserved in ovary compared to testis when comparing Italian sparrow with parental species. However, having only reproductive tissue, the effects of sex and tissue are intertwined, and it is plausible that some of the differences between ovary and testis are due to physiological differences between sexes. In both ovary and testis, we observed fewer differentially expressed genes in comparison of Italian sparrow to house than to Spanish sparrow. In the ovary, while the expression pattern in Italian sparrow is house sparrow‐dominant, it is important to also consider that the smaller sample size of Spanish sparrow ovary (*n* = 3) might have led to an over‐estimation of differentially expressed genes. In testis, we found over‐expression of genes involved in functions related to mitochondrial respiratory chain, cytosolic and mitochondrial ribosomal proteins in the hybrid Italian sparrow. Mito‐nuclear interactions can strongly affect gene expression (Sanchez‐Ramirez et al., 2021), and even result in mis‐expression (Runemark, Eroukhmanoff, et al., 2018). Mito‐nuclear genes have a role in reproductive isolation in Italian sparrows and have been under selection for inheritance of the house sparrow allele (Hermansen et al., 2014; Runemark, Trier, et al., 2018; Trier et al., 2014). Correct mitochondrial function requires a coordinated expression of both nuclear and mitochondrial genomes (Ryan & Hoogenraad, 2007). Therefore, the upregulation of genes involved in respiratory chains and protein synthesis corroborate the evidence for a strong effect of hybridization on genes associated with metabolism in other bird taxa (Wagner et al., 2020). Overexpression of cytosolic ribonucleoproteins and mitochondrial respiratory chain enzymes has also been observed in later generation hybrids (F_3+_) in copepod species and has been linked to cellular responses to physiological dysfunction (Barreto et al., 2015). Our study thus contributes to the growing body of evidence of mito‐nuclear interactions as important reproductive barriers (Hill, 2017), and provides insight into altered patterns of gene expression as a possible resolution to the conflict.

In addition to mito‐nuclear genes, over‐dominant genes in Italian sparrow contained ribonucleoproteins present in both cytosolic and mitochondrial ribosomes. RNA binding proteins play an important role in post‐translational regulation of gene expression and spermatogenesis and appear to be key regulatory factors that ensure male fertility (Paronetto & Sette, 2010; Phillips et al., 2019). Several of the over‐dominant genes also coded for subunits of T‐complex protein Ring Complex (TRiC) involved in folding of about 10% of the proteome. Subunits of TRiC are required for spermatogenesis (Counts et al., 2017) and are under positive selection among the seminal fluid genes in passerine species (Rowe et al., 2020).

Although we suggest caution in interpretation of our experimental F_1_ hybrid data due to their unusual genomic composition, our work is in agreement with findings from F_1_s in two other songbird species (Davidson & Balakrishnan, 2016; Mugal et al., 2020). F_1_ hybrids of flycatchers also showed a tissue‐specific pattern of expression differences, with a low degree of transgression in genes expressed in testis (Mugal et al., 2020), similar to the pattern in our experimental hybrids but contrasting with the strong transgression in the testis transcriptome of the Italian sparrow. Future studies should therefore extend sampling to nonreproductive tissues to assess whether transgressive expression is consistent across tissues. While a standardized environment and time for sampling would be ideal, this is not easily achieved in wild species that differ in geographical distributions, phenologies and thermal comfort zones, as sampling at the same time could capture different phenological stages. Future studies should sample these species over time to remedy this complication. In addition, wild‐caught individuals might have a higher within‐group variation, and when compared to captive birds, it is critical for them to be held at similar environmental conditions before sample collection. A final aspect that would be interesting to investigate in the future is to assess whether cell composition in testis differs between species and potentially contributes to differences in gene expression (Good et al., 2010; Hunnicutt et al., 2022). Since all species are fertile, we do not expect differences in cell composition in this study, but single‐cell transcriptomics could be used to confirm this.

Homoploid hybrid species are expected to have higher levels of genetic variance due to homologous recombination leading to transgressive phenotypes (Mallet, 2007; Rieseberg et al., 2003). The extensive level of transgressive expression in the Italian sparrow testis is therefore in agreement with this expectation. Gene expression is the key intermediate step between genotype and phenotype, so examining the link between transgressive gene expression and transgressive hybrid phenotypes is a crucial next step, which will significantly increase our understanding of how novel variation arises from hybridization and the role of gene expression divergence in the evolution of reproductive isolation from parental species.

## AUTHOR CONTRIBUTIONS

A.R. and H.P.Y. conceived the study. A.M. and S.M. raised the experimental F_1_ hybrids and M.Ro., M.Ra., A.R., F.E., C.O.G. and G‐P.S. performed field work and sampling, C.O.G performed the molecular laboratory work, and H.P.Y. designed and performed the bioinformatical analysis based on discussions with A.R., M.Ra. and M.Ro. H.P.Y. drafted the manuscript, with revisions based on input from all co‐authors.

## CONFLICT OF INTERESTS

The authors declare that they have no conflict of interest.

### OPEN RESEARCH BADGES

This article has earned an Open Data Badge for making publicly available the digitally‐shareable data necessary to reproduce the reported results. The data is available at BioProject PRJNA832330. https://(github.com/Homap/Expression_sparrow).

## BENEFIT‐SHARING STATEMENT

Research was performed in collaboration and cooperation with local researchers from the University of Extremadura. All collaborators are included as co‐authors. International collaboration has been fundamental to this work and has contributed to sharing of scientific results, cooperation, education and training.

## Supporting information


Appendix S1

## Data Availability

Raw RNA sequencing reads are deposited in the NCBI Short Read Archive (BioProject PRJNA832330). R script containing code for differential gene expression analyses and the figures presented in this paper are deposited under github.com/Homap/Expression_sparrow. In this work, all samples taken from Italy, Spain and Oslo were in accordance with the Nagoya protocols. In Spain, all trapping and sampling of birds were conducted in accordance with Spanish Animal Protection Regulation RD53/2013, and all methods were approved by the Institutional Commission of Bioethics at the University of Extremadura (CBUE 49/2011). Birds were sampled in Oslo with permission from Miljø‐direktoratet (2016/2225). In Italy, all birds were sampled with permission from ISPRA (protocol no. 12404) and with permission (no. 305) from the Puglia region.
